# Rotavirus Recombinant VP6 Nanotubes Act as an Immunomodulator and Delivery Vehicle for Norovirus Virus-Like Particles

**DOI:** 10.1155/2016/9171632

**Published:** 2016-09-04

**Authors:** Maria Malm, Kirsi Tamminen, Suvi Lappalainen, Timo Vesikari, Vesna Blazevic

**Affiliations:** Vaccine Research Center, University of Tampere, Biokatu 10, 33520 Tampere, Finland

## Abstract

We have recently shown that tubular form of rotavirus (RV) recombinant VP6 protein has an* in vivo* adjuvant effect on the immunogenicity of norovirus (NoV) virus-like particle (VLP) vaccine candidate. In here, we investigated* in vitro* effect of VP6 on antigen presenting cell (APC) activation and maturation and whether VP6 facilitates NoV VLP uptake by these APCs. Mouse macrophage cell line RAW 264.7 and dendritic cell line JAWSII were used as model APCs. Internalization of VP6, cell surface expression of CD40, CD80, CD86, and major histocompatibility class II molecules, and cytokine and chemokine production were analyzed. VP6 nanotubes were efficiently internalized by APCs. VP6 upregulated the expression of cell surface activation and maturation molecules and induced secretion of several proinflammatory cytokines and chemokines. The mechanism of VP6 action was shown to be partially dependent on lipid raft-mediated endocytic pathway as shown by methyl-*β*-cyclodextrin inhibition on tumor necrosis factor *α* secretion. These findings add to the understanding of mechanism by which VP6 exerts its immunostimulatory and immunomodulatory actions and further support its use as a part of nonlive RV-NoV combination vaccine.

## 1. Introduction

Noroviruses (NoVs) and rotaviruses (RVs) are the major causative agents of pediatric acute gastroenteritis (AGE) in children worldwide. There is no licensed vaccine for NoV available, and despite efficacious live RV vaccines currently in use [1], there is a need [2] for nonlive RV vaccines that could be safer [3], more affordable, and more efficacious in developing countries [4–6]. Our group has developed a subunit combination vaccine candidate against NoV and RV gastroenteritis, consisting of NoV virus-like particles (VLPs) and RV VP6, aiming to confer protection from both leading etiological agents of severe AGE [7–9].

The double-stranded (ds) RNA genome of triple-layered RV particle is surrounded by core protein VP2 [10]. The outermost layer, composed of VP7 capsid glycoprotein and the spike protein VP4, contains most epitopes for RV neutralizing antibody interaction [11]. The intermediate layer is formed by the major internal structural protein VP6 that represents 51% of the virion mass [12]. VP6 trimers are organized into hexagons and packed into higher order structures, for example, nanotubes, nanospheres, or sheets when expressed* in vitro* in baculovirus (BV) or bacterial expression systems [12–16]. The variable morphology of polymeric VP6 protein expressed* in vitro* depends on biochemical composition, mainly on pH and ionic strength [17].

Recombinant VP6 has been considered as a nonlive next generation vaccine candidate against RV by us and others, being the most abundant, highly conserved, and immunogenic RV protein [2, 7, 8, 18]. B-cell-mediated immune responses, especially IgA seroconversion following RV vaccination and natural infection, are mostly directed against VP6 [19]. Even though the inner capsid protein VP6 cannot elicit classical neutralizing antibodies, it induced heterotypic protective immunity against live RV challenge in mice that correlated with postchallenge VP6-specific serum IgA [20]. VP6-specific polymeric IgA inhibits RV replication intracellularly during IgA transcytosis, a phenomenon termed intracellular neutralization [21–23].

We have recently reported that VP6 protein provides dose sparing effect on NoV VLPs* in vivo* and acts as both Th1 and Th2 type adjuvant on immune response induced by NoV VLPs [24]. Other investigators have shown that when VP6 is used as an antigen carrier or delivery platform for foreign antigens, the response to the antigen was improved [25, 26]; however, the mechanism was not investigated. Particulate nature and characteristics of an antigen, such as size, shape, surface charge, and receptor interactions, are generally considered important for antigen presenting cell (APC) targeting [27]. Moreover, the antigen size influences development and quality of immune responses [28, 29]. Even though VP6 nanotubes and RV double-layered (dl) 2/6-VLPs have been shown to be equally immunogenic* in vivo* [30], there have also been indications of improved uptake of VP6 nanotubes compared to spherical forms of RV dlVLP [31].

We hypothesized that due to their size and morphology VP6 nanotubes may be potent inducers of APC activation and maturation. APCs are known to be critical for initiating and modulating antigen-specific immune responses [32, 33] and therefore are likely to have a key role in VP6 exerted adjuvant effect [24, 31]. In here, we investigated the effect of VP6 nanotubes on two commonly used APC lines, RAW 264.7 macrophages and JAWSII immature dendritic cells (DCs), aiming to improve understanding of the immunostimulatory and immunomodulatory mechanism of VP6 oligomeric protein.

## 2. Materials and Methods

### 2.1. Cell Lines, Viruses, and Culture Conditions

Adherent murine immortalized RAW 264.7 macrophage cell line, originating from blood monocyte/macrophages (H-2d) (Cat. TIB71), and JAWSII immature DCs, derived from bone marrow of H-2b C57BL/6 p53-knockout mouse (Cat. CRL-11904) [34], were obtained from the American Type Culture Collection (ATCC®, Manassas, VA). The cells were grown at 37°C in a 5% CO_2_ air humidified atmosphere and used for the treatments after at least 5 but less than 20 passages in our laboratory. RAW 264.7 cells were cultured in Dulbecco's modified Eagle's minimum essential medium (DMEM, Cat. D6546), supplemented with heat inactivated 10% fetal bovine serum (FBS, Cat. F9665), 100 units/mL penicillin, 100 *μ*g/mL streptomycin (Cat. P0781), and 2 mM L-glutamine (Cat. G7513) (all from Sigma, St. Louis, MO). The JAWSII cells were maintained in minimum essential medium Eagle, alpha minimum essential medium (*α*MEM) supplemented with ribonucleosides, deoxyribonucleosides (Cat. M8042, Sigma), 1 mM sodium pyruvate (Cat. S8636, Sigma), 20% FBS, 4 mM L-glutamine, and 5 ng/mL recombinant murine granulocyte-macrophage colony-stimulating factor (GM-CSF, Cat. 415ML-010, R&D Systems, Inc.). Human epithelial colorectal adenocarcinoma cell line Caco-2 (ATCC, Cat. HTB-37) was used for internalization studies. Adherent Caco-2 cell line was cultured in a complete culture medium consisting of DMEM medium, 10% FBS, 100 units/mL penicillin, and 100 *μ*g/mL streptomycin. Caco-2 cells were subcultured when they reached ~80% confluency and were not allowed to form tight monolayer or to differentiate before being used for the internalization analyses. Human RV strain Wa (G1P1A [8]) was propagated in fetal rhesus monkey kidney (MA104) cells as described elsewhere [30].

### 2.2. Recombinant Proteins Production and Purification

**Figure 1:**
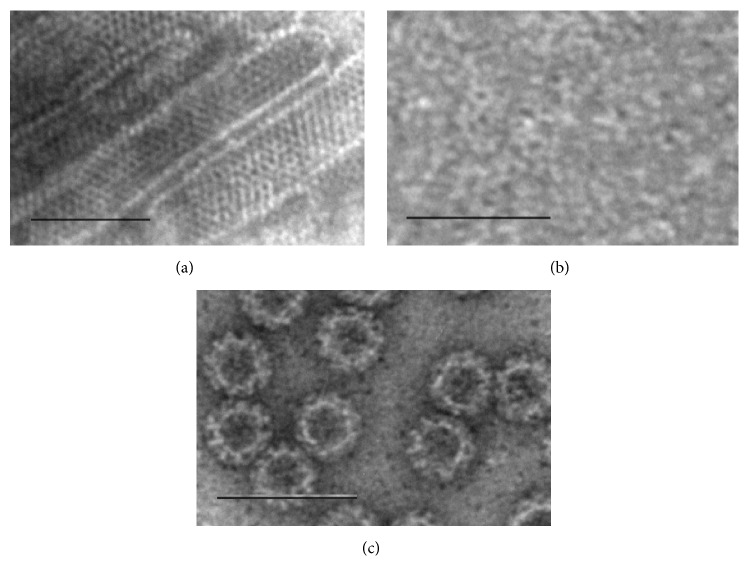
Structure and integrity of the proteins. Electron microscopy images of RV VP6 nanotubes (a), trimeric VP6 (b), and NoV GII.4 VLPs (c). Protein products were examined by FEI Tecnai F12 electron microscope (Philips Electron Optics, Holland) following negative staining with 3% uranyl acetate, pH 4.6. Bar 100 nm. Images observed at 9300x (a and b) and 11,000x (c) magnification.

Human RV VP6 protein (database accession number GQ477131) and NoV GII.4 (reference strain accession number AF080551) VLPs were produced in a BV-insect cell expression system according to the previously described procedures [7, 8, 35]. The proteins were highly purified with demanding multistep chromatographic procedures [24] and the concentration, purity, integrity, and morphology of the proteins were verified as described in detail elsewhere [7, 24]. In brief, Pierce BCA protein assay (Thermo Scientific, Cat. 23227) was used for quantification of the total protein. The integrity of RV VP6 nanotubes (Figure 1(a)), trimeric VP6 (Figure 1(b)), and NoV GII.4 VLPs (Figure 1(c)) was verified under electron microscopy (EM). The purity was confirmed by Quant-it dsDNA Broad-Range Assay Kit (Invitrogen, Cat. Q33120; <10 ng dsDNA/10 *μ*g of protein) and sodium dodecyl sulfate polyacrylamide gel electrophoresis (SDS-PAGE) and densitometric analysis after silver staining (PageSilver*™* silver staining kit, Fermentas, Cat. K0681) (data not shown). Infectious BV and endotoxin level were determined with BacPAK*™* RapidTiter Kit (Clontech Laboratories, Cat. 631406; 0 pfu live BV/mL) and Limulus Amebocyte Lysate assay (Lonza, Cat. 244 N184-25; <0.1 endotoxin units/100 *μ*g of protein). Crude purified VP6 preparation containing impurities related to the expression system, for example, residual BVs (10^6^ plaque forming units/mL), was used in parallel with pure VP6 in internalization inhibition experiments as described below. The proportion of VP6 in impure VP6 preparation was calculated after the densitometric analysis of PAGE with AlphaEase® FC Software (Alpha Innotech, San Leandro, CA), as described previously [30].

### 2.3. Internalization of RV VP6, RV Wa, and GII.4 VLPs by Cell Lines

Uptake of RV VP6 nanotubes and RV Wa was tested in mouse RAW 264.7 macrophages, JAWSII DCs, and human colorectal epithelial Caco-2 cell lines. Each cell line was plated to cell-culture treated multidish wells and was allowed to attach overnight in the cell-type-specific culture medium. The culture medium was replaced with the medium containing 100 *μ*g/mL of highly purified VP6 nanotubes, 1 : 10 diluted RV Wa, or fresh culture medium (for untreated control cells). After the incubation period of 4 h for RAW 264.7 cells only or 24 h for all three cell types, the cells were harvested and supernatants collected and stored at −80°C for cytokine analysis. The VP6 uptake was analyzed by intracellular staining and flow cytometry as described below.

The internalization of NoV GII.4 VLPs and cointernalization of GII.4 VLPs with RV VP6 nanotubes were carried out using the RAW 264.7 cells and 24 h incubation. Cells were pulsed with 100 *μ*g/mL GII.4 alone or 100 *μ*g/mL of GII.4 combined with 100 *μ*g/mL of VP6. The internalization of GII.4 VLPs was analyzed by intracellular staining of NoV VLP and flow cytometry.

### 2.4. Macrophage and DC Activation and Maturation Analysis

VP6 mediated activation and maturation were explored by determining the change in the expression of cell surface markers and upregulation of cytokine secretion. After overnight incubation of RAW or JAWSII cells on the plates, the cell-culture medium was changed and cells were exposed to RV VP6 nanotubes (10 *μ*g/mL or 50 *μ*g/mL), trimeric (denatured) VP6 (10 *μ*g/mL or 50 *μ*g/mL), GII.4 VLPs (10 *μ*g/mL or 50 *μ*g/mL), RV Wa (1 : 10 dilution), or 1 *μ*g/mL of bacterial lipopolysaccharide (LPS) (Cat. L6143, Sigma) and incubated further for 24 h or 48 h. Supernatants of all cultures were collected, centrifuged, and stored at −80° until cytokine analysis. The cell surface expression of costimulatory and maturation markers CD80, CD86, CD40, and major histocompatibility complex (MHC) class II molecules was analyzed by flow cytometry as described below.

### 2.5. Inhibition of RV VP6 Internalization

Pharmacological internalization inhibitors methyl-*β*-cyclodextrin (M*β*CD, Cat. C4555), chloroquine diphosphate salt (Cat. C6628), and cytochalasin D from* Zygosporium mansonii* (Cat. C2618) (all from Sigma) were employed. RAW cells were plated and allowed to attach before adding the inhibitors at predetermined nontoxic concentrations. Cells were preincubated in cell-culture medium supplemented with 15 *μ*M chloroquine, 2 mM M*β*CD (without serum), or 2 *μ*M cytochalasin D for 60 min before exposing cells to 50 *μ*g/mL of VP6 nanotubes and incubating them further for 17 h. In addition, the concentration of 50 *μ*g/mL of crude purified VP6 nanotubes containing impurities related to the expression system, for example, residual BVs, was used as a control.

### 2.6. Flow Cytometry Analysis

The cells were harvested and washed with cold PBS + 3% FBS. Prior to staining or permeabilization the cells were blocked with rat anti-mouse CD16/CD32 (Fc Block, Clone 2.4G2, Becton Dickinson, San Jose, CA) on ice for 10 min followed by washing with PBS + 3% FBS. For intracellular staining of the internalized RV VP6 or NoV GII.4 VLPs, the BD Cytofix/Cytoperm Plus kit (Becton Dickinson, San Jose, CA) was used according to manufacturer's instructions. For detection of VP6, permeabilized and fixed cells were stained intracellularly with rabbit polyclonal rotavirus group A antibody (Cat. GWB-459FC9, Genway Biotech Inc.) followed by fluorescein isothiocyanate- (FITC-) conjugated goat anti-rabbit Ig (BD Pharmingen). For GII.4 VLP staining, a human serum highly positive for anti-GII.4 NoV antibodies was used at 1 : 2000 dilution, followed by polyclonal anti-human IgG (Fc *γ*-specific) phycoerythrin (PE) conjugate (eBioscience) for detection. Internalization was examined by overlaying the histograms of treated and untreated cells and by comparing mean fluorescence intensity (MFI) of each population.

For the cell surface molecules expression analysis, RAW 264.7 cells were stained on ice for 30 min with anti-mouse monoclonal antibodies PerCP-Cy5.5-conjugated CD80 (16-10A1), PE-Cy7-conjugated CD86 (GL1), PE-conjugated CD40 (3/23), and Alexa Fluor 647-conjugated I-A/I-E (M5/114.15.2) (mouse MHC class II). BD CompBeads used for compensation and conjugated cell surface marker monoclonal antibodies were purchased from BD Biosciences.

The flow cytometry analyses were performed on a FACSCanto*™* II (Becton Dickinson, San Jose, CA) flow cytometer, using the FACSDiva*™* Version 6.1.3 software (Becton Dickinson). The data analysis was carried out with FlowJo software version 10 (Three Star Inc., San Carlos, CA).

### 2.7. Cytokine ELISAs

Quantities of tumor necrosis factor alpha (TNF-*α*), interleukin-6 (IL-6), and interferon-alpha (IFN-*α*) cytokines in RAW and JAWSII cell supernatants were determined by commercial enzyme-linked immunosorbent assay (ELISA) kits: mouse TNF-*α* DuoSet (Cat. DY410-05), mouse IL-6 DuoSet (Cat. DY406-05), and VeriKine mouse IFN alpha ELISA kit (Cat. 42120-1, PBL Assay Science) according to the manufacturers' instructions. Victor^2^ 1420 Multilabel Counter (Wallac, Perkin Elmer) plate reader was used for optical density reading (OD) of the plate. For each assay the background signal from the blank wells (wells without supernatant) was subtracted from all of the OD readings on the plate. Standard curves were plotted and used for calculating the cytokine concentration of each sample (pg/mL).

### 2.8. Cytokine Array

Cytokine antibody array was performed with a mouse cytokine array kit (Cat. ARY006, R&D Systems, Inc., MN) according to the manufacturer's protocol. Briefly, RAW 264.7 cell-culture supernatants were collected after 24 h stimulation with 100 *μ*g/mL VP6 nanotubes or left untreated. Supernatants were centrifuged and stored at −80°C until analyzed. The membranes precoated with 40 different cytokine/chemokine-specific capture antibodies were blocked and incubated with 1 : 5 diluted supernatants overnight at +4°C. After washing, the detection antibody cocktail was added for 30 min, followed by streptavidin-horseradish peroxidase and chemiluminescent detection reagent. The immunoblot images were captured and visualized using a Bio-Rad ChemiDoc*™* MP system and the intensity of each spot in the captured images was analyzed using a ImageJ 1.50b software.

### 2.9. Statistical Analysis

Cytokine release (pg/mL) in ELISA was compared using Mann–Whitney *U*  test. The data were analyzed by IBM SPSS Statistics software version 22.0 and *p*  value of <0.05 was considered statistically significant.

## 3. Results

### 3.1. Internalization of VP6 Nanotubes by Macrophages and Immature DC

**Figure 2:**
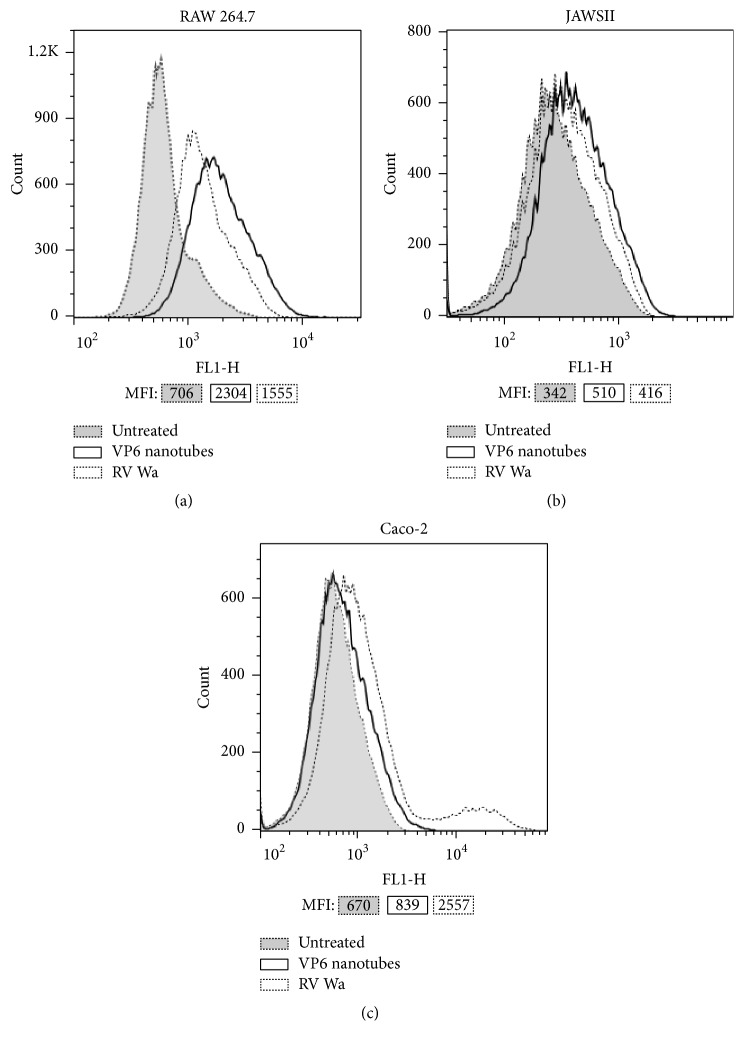
Internalization of VP6 nanotubes. Flow cytometry analysis of VP6 uptake by RAW 264.7 macrophages (a), JAWSII DCs (b), and human colorectal Caco-2 cells (c) after 24 h incubation in the presence of 100 *μ*g/mL VP6. Cells exposed to rotavirus (RV) Wa and untreated cells incubated in culture medium only served as controls. VP6 was detected by intracellular staining with rabbit anti-rotavirus antibody followed by goat fluorescein isothiocyanate-conjugated anti-rabbit Ig antibody. Shown are overlaid histograms of cells exposed to VP6 nanotubes or RV Wa and untreated cells of one representative experiment of three independent experiments performed. Mean fluorescence intensity (MFI) values of each histogram are indicated.

After incubating RAW 264.7, JAWSII, or human colorectal epithelial Caco-2 cells with 100 *μ*g/mL of VP6 or RV Wa, the entry of the RV protein was examined by flow cytometry and compared to cells incubated with culture medium only (untreated cells). VP6 nanotubes were shown to be internalized by macrophages (Figure 2(a)) and DC (Figure 2(b)) but not by Caco-2 epithelial cells (Figure 2(c)).

Within 24 h of incubation of RAW cells with VP6, the RV-specific intracellular staining increased by 202–256% (range of percent increase of MFI for three independent experiments) compared to untreated cells. A 120% increase was observed in RAW cells exposed to RV Wa (Figure 2(a)). VP6 entry to RAW cells was observed already at 4 h but at a much lower quantity than after 24 h (data not shown). VP6 was also internalized by JAWSII cells within 24 h incubation but less efficiently than by RAW macrophages (Figure 2(b)). Compared to untreated cells, VP6 treatment induced a moderate 49–60,9% increase in RV-specific intracellular staining. In contrast to these results, a control cell line for internalization, human colorectal epithelial Caco-2 cells highly susceptible to RV infection [36], did not take up VP6 alone, but a 282–311% increase in MFI was detected after RV Wa exposure (Figure 2(c)).

### 3.2. Activation and Maturation Phenotype of APCs Induced by VP6 Nanotubes

**Figure 3:**
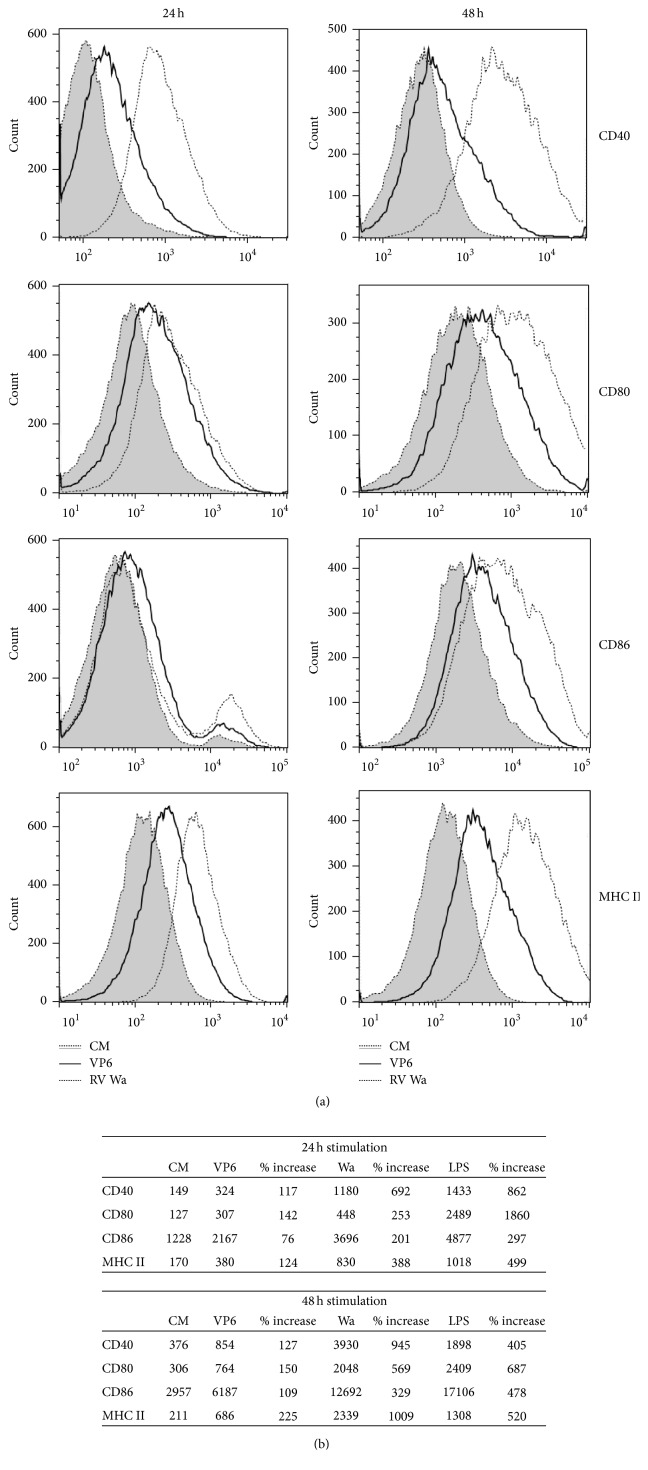
Effect of VP6 nanotubes on RAW 264.7 cell surface markers expression. The expression of CD40, CD80, CD86, and MHC class II (I-A/I-E) was analyzed by flow cytometry after 24 h and 48 h incubation in the presence of 50 *μ*g/mL VP6, 1 : 10 diluted RV Wa, 1 *μ*g/mL LPS, or culture media (CM) only. (a) Expression of the molecules on the cells in CM only and upregulation induced by VP6 and RV Wa are shown by overlaid histograms. The results are representative of three independent analyses done. (b) Table of mean fluorescence intensity (MFI) values obtained from the histograms in Figure 3(a) of each cell surface marker after treatment with VP6, RV Wa, LPS, or untreated cells.

After 24 h and 48 h stimulation of RAW and JAWSII cells with 50 *μ*g/mL of VP6 nanotubes, 1 : 10 diluted RV Wa, or 1 *μ*g/mL of LPS, the change in the expression of cell surface molecules CD80, CD86, CD40, and MHC II was examined by flow cytometry. As shown in Figures 3(a) and 3(b), VP6 stimulation upregulated MFI of all four markers compared to untreated RAW cells already in 24 h, which further increased within 48 h. LPS, used as a positive control, upregulated strongly all markers already within 24 h as expected (Figure 3(b)). Viral control (RV Wa) also induced high surface marker upregulation similarly to LPS (Figures 3(a) and 3(b)). Similar pattern of cell surface molecules expression upregulation with all three stimuli was observed with JAWSII cells but the increases were not as prominent as with the RAW cells (data not shown).

### 3.3. Effect of VP6 on Proinflammatory Cytokines and Chemokine Secretion

**Figure 4:**
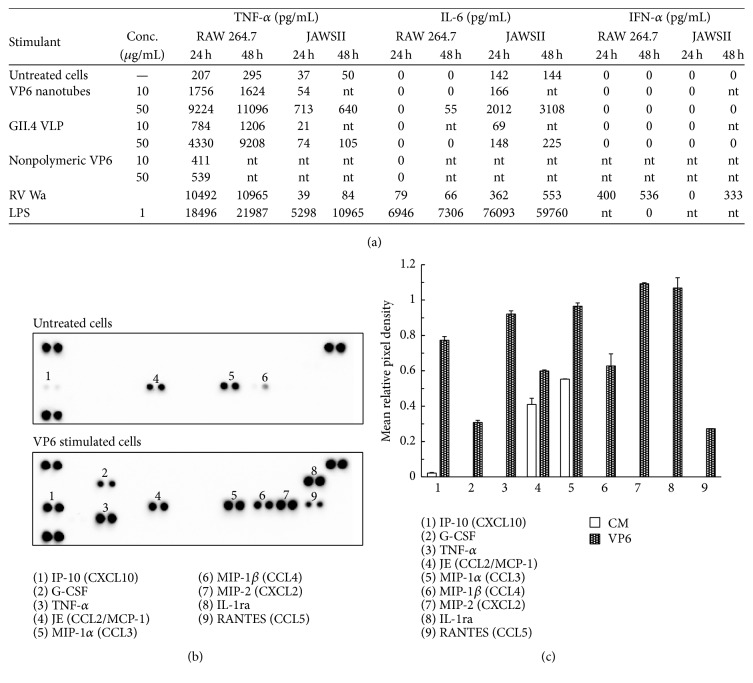
Cytokine production in the supernatants of RAW 264.7 and JAWSII cells. (a) The quantity (pg/mL) of TNF-*α*, IL-6, and IFN-*α* in the supernatants of RAW and JAWSII cells incubated for 24 h or 48 h with 10 *μ*g/mL and 50 *μ*g/mL of VP6 nanotubes, NoV GII.4 VLPs, or nonpolymeric VP6. RV Wa, LPS, and culture media only (untreated cells) served as controls. Zero values indicate that the result was below the lowest standard dilution of the assay (62.5 pg/mL for TNF-*α*, 15.6 pg/mL for IL-6, and 12.5 pg/mL for IFN-*α*). nt = not tested. Shown are the mean values of analysis done at least twice with independent samples. (b) The mouse cytokine multiplex array analysis of RAW 264.7 supernatants after 24 h stimulation with VP6 nanotubes (100 *μ*g/mL) or fresh culture media (untreated cells). (c) The pixel densities ± SD of the replicates for each visible spot on the arrays, calculated using ImageJ software and plotted.

After 24 h or 48 h incubation of RAW and JAWSII cells with VP6 nanotubes, GII.4 VLPs, nonpolymeric denatured VP6 (10 *μ*g/mL and 50 *μ*g/mL each protein), RV Wa, or LPS, supernatant was collected and TNF-*α*, IL-6, and IFN-*α* were quantified by ELISA (Figure 4(a)). Nonpolymeric trimeric VP6 lacking conformational structures (Figure 1(b)) was used as a control protein.

Both RAW and JAWSII cells secreted TNF-*α* in a dose-response manner after 10 and 50 *μ*g/mL VP6 nanotube exposure (Figure 4(a)). However, GII.4 VLP did not induce considerable TNF-*α* in JAWS cells (74 ± 57 pg/mL, resp.) as did VP6 nanotubes at the same concentration (713 ± 296 pg/mL). TNF-*α* production was significantly higher in VP6 nanotube exposed cells in both RAW and JAWSII cells compared to untreated cells (*p* < 0.05). These two cell lines exhibited different cytokine expression profiles upon stimulation, the primary cytokine secreted by RAW cells being TNF-*α*, while JAWSII DCs produced higher quantities of IL-6 (Figure 4(a)). Interestingly, VP6 nanotubes and RV Wa induced similar TNF-*α* secretion in RAW cells (*p* > 0.05), but only VP6 stimulated significant production of TNF-*α* in JAWSII cells. A tubular structure of VP6 was essential for the stimulatory effect of the protein, as more TNF-*α* was secreted after exposing RAW cells to 10 *μ*g/mL (1756 ± 135 pg/mL) or 50 *μ*g/mL (9224 ± 1409 pg/mL) of VP6 nanotubes, compared to the equal concentrations of nonpolymeric VP6 control (411 ± 75 and 539 ± 14 pg/mL, resp.).

The constitutive IL-6 secretion by JAWSII cells was remarkably stimulated by VP6 nanotubes, whereas only subtle changes were detected in the supernatants of the cells incubated in the presence of GII.4 VLP (Figure 4(a)). RV Wa had a moderate effect on IL-6 secretion by JAWSII cells, whereas the positive control LPS elicited high IL-6 secretion. Only small quantities of IL-6 were produced by RAW cells stimulated with VP6 and RV Wa but not at all with GII.4 VLPs. As expected, antiviral cytokine IFN-*α* secretion was detected only after RV Wa stimulation in both cell lines but not when exposed to protein products or LPS.

The cytokine profile of RAW supernatant stimulated with 100 *μ*g/mL VP6 for 24 h was further examined by cytokine array, indicating a strong activation of the cells (Figures 4(b) and 4(c)). Several cytokines and chemokines were upregulated (Figure 4(c)), including interferon gamma-inducible protein (IP-10) and macrophage inflammatory protein (MIP-) 1*α* and MIP-1*β*, when compared to supernatant of untreated cells (CM only). However, TNF-*α*, granulocyte-colony-stimulating factor (G-CSF), MIP-2, IL-1ra, and RANTES were only detected in VP6 stimulated cells. As IL-6 cytokine production by RAW cells at 24 h was not detected by ELISA (Figure 4(a)) and it was not detected by the cytokine array either, corroborating the findings (data not shown).

### 3.4. Effect of Chemical Inhibitors on Internalization and TNF-*α* Production

**Figure 5:**
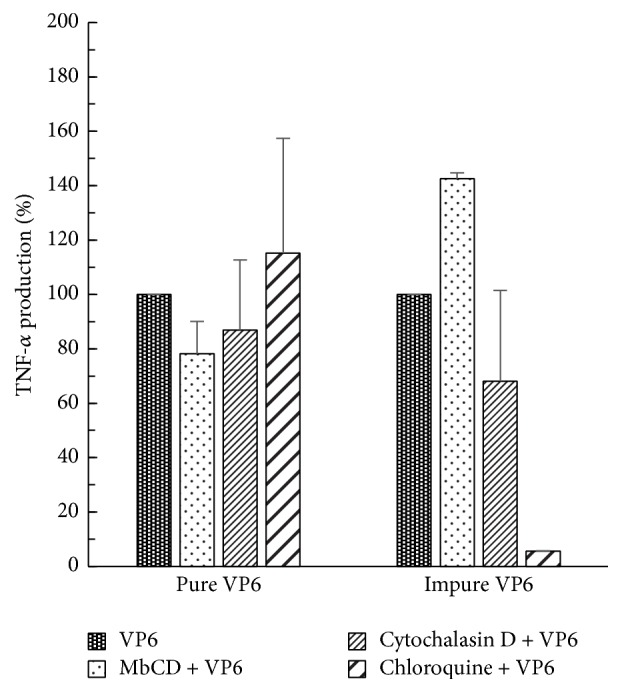
Effect of chemical inhibitors on VP6-induced TNF-*α* secretion by RAW 264.7 cells. The change in TNF-*α* production (%) by pharmacological inhibitors M*β*CD, cytochalasin D, or chloroquine of cells stimulated with 50 *μ*g/mL pure or impure VP6 nanotubes for 17 h in the presence or absence of an inhibitor. VP6-induced TNF-*α* production (pg/mL) without an inhibitor was considered 100%. Shown are the mean values of two independent analyses with standard deviations.

The effect of chemical inhibitors M*β*CD, cytochalasin D, and chloroquine on the VP6 nanotube internalization and VP6-induced TNF-*α* secretion by RAW 264.7 cells was investigated. Both highly purified VP6 nanotubes and the VP6 nanotubes known to contain BV-related impurities, which assumingly employ different entry pathways into the cells, were used in these experiments. After M*β*CD treatment, a 22 ± 11% decrease in TNF-*α* secretion was observed in RAW cells stimulated with pure VP6 (Figure 5), whereas M*β*CD showed an increasing effect (43 ± 2%) when impure VP6 was used. Other two chemical inhibitors, cytochalasin D and chloroquine, did not decrease TNF-*α* production in RAW cells induced by pure VP6 nanotubes. On the contrary, TNF-*α* production induced by impure VP6 was almost completely blocked (94 ± 0.1%) by chloroquine treatment (Figure 5), indicating that the two protein preparations have different stimulatory mechanism. This observation was further supported by ~26% inhibition of impure VP6 internalization by chloroquine treatment, while the internalization of pure VP6 did not significantly change with any of the treatments (data not shown).

### 3.5. Increased GII.4 VLP Uptake and APC Activation and Maturation by VP6 Nanotubes Codelivery

**Figure 6:**
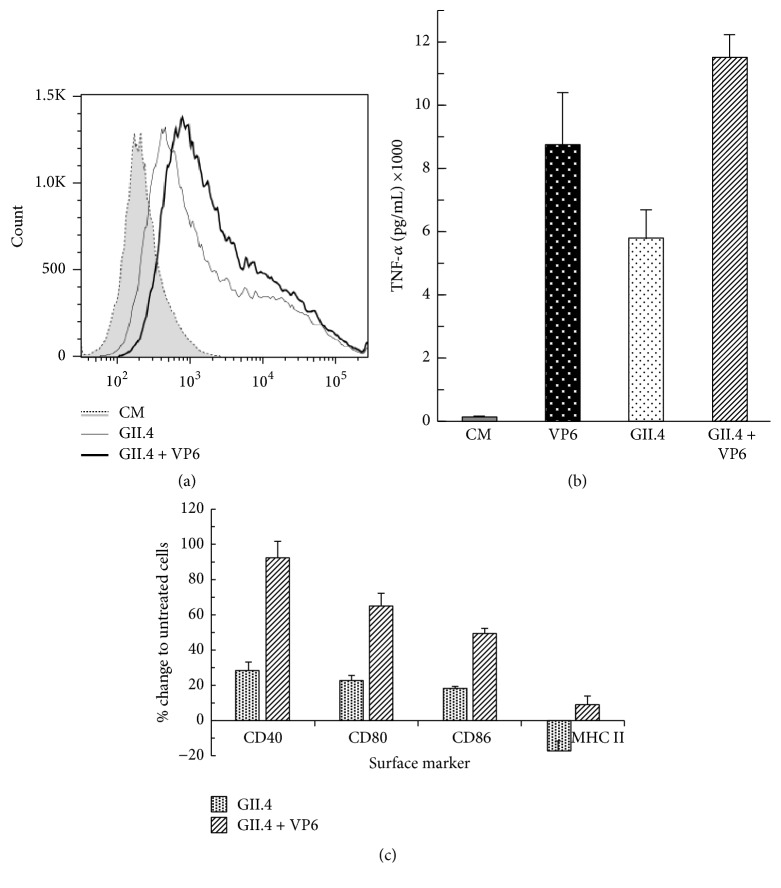
Internalization and activating/maturating effect of NoV GII.4 VLPs alone or combined with RV VP6 nanotubes on RAW 264.7 cells. (a) Flow cytometry analysis of GII.4 VLPs uptake by RAW 264.7 cells after 24 h incubation with 100 *μ*g/mL of GII.4 VLPs alone or mixed with 100 *μ*g/mL of VP6 nanotubes. The intracellular staining of untreated cells (CM), GII.4 VLP treated cells, and GII.4 + VP6 treated cells, of a representative experiment of two independent analyses, is shown. (b) The level of TNF-*α* in cell-culture supernatants as measured by ELISA after 24 h incubation with 100 *μ*g/mL of GII.4 VLPs alone, 100 *μ*g/mL VP6 alone, or GII.4 VLPs mixed with 100 *μ*g/mL of VP6 nanotubes. Shown are the mean values and standard deviations of two independent analyses. (c) The expression of cell surface markers measured by flow cytometry after 24 h stimulation. The results represent % increase or decrease of the MFI of each marker on the cells incubated with GII.4 VLPs alone or with combined GII.4 VLPs and VP6 nanotubes compared to baseline level of untreated cells. The mean values with standard deviations of two independent analyses are presented.

The coadministration of VP6 nanotubes with GII.4 VLPs improved uptake of GII.4 VLPs to RAW 264.7 cells (Figure 6(a)). When RAW cells were incubated for 24 h with 100 *μ*g/mL GII.4 VLPs, 47% of the cells were positive for NoV GII.4 when analyzed by intracellular staining. Coadministration of GII.4 VLPs with VP6 nanotubes further increased the number of NoV-positive cells by 30% (from 47% to 61%, resp.). A twofold increase in TNF-*α* secretion was observed in cells incubated with GII.4 + VP6 combination (11490 ± 741 pg/mL), compared to GII.4 VLPs alone (5761 ± 922 pg/mL) (Figure 6(b)), indicating an additive effect of the two proteins on the cytokine secretion. Furthermore, improved maturation of RAW macrophages was achieved with coadministration, as illustrated in Figure 6(c). NoV GII.4 VLPs + VP6 upregulated cell surface molecules CD40, CD80, CD86, and MHC II expression compared to GII.4 VLPs alone. Interestingly, MHC II downregulation was repeatedly observed when incubating RAW cells with GII.4 VLPs only.

## 4. Discussion

We have recently shown* in vivo* adjuvant effect of VP6 nanostructures on the immunogenicity of NoV VLPs [24], where coadministration of VP6 nanotubes with suboptimal doses of NoV GII.4 VLPs increased the level of type-specific and cross-reactive NoV-specific immune responses. Therefore, in the present work, we investigated the effect of VP6 on activation and maturation of mouse RAW 264.7 macrophages and JAWSII immature DCs. Macrophages and DCs are professional APCs with high endocytic capacity known to be central for mounting effective T- and B-cell immune responses to foreign antigens* in vivo* [37–40]. The results provide an insight into the mechanism of VP6 adjuvant action.

RAW and JAWSII cells were first tested for uptake of VP6* in vitro*. Extremely pure recombinant VP6 nanotubes were efficiently internalized by the RAW macrophages and to less extent by JAWSII DCs. Human epithelial Caco-2 cells were used as a control cell line as, unlike APCs, they are not efficient in uptake of larger particles but are susceptible to human RV Wa infection [36]. Accordingly, no significant uptake of VP6 was detected in Caco-2 cells, while RVs were readily internalized. These results are congruent with the report by Rodríguez et al. [31], who showed that VP6 nanotubes were favorable for macrophage uptake.

Along with being internalized by macrophages and immature DCs, VP6 nanotubes induced activation and maturation of APCs by upregulating cell surface costimulatory and antigen presenting molecules CD40, CD80, CD86, and MHC II. Signaling through CD80 and/or CD86 on mature APCs, which bind to CD28 on T cells [41, 42], is a key costimulatory pathway for antigen-specific T cell activation. A bacterial activator LPS, a toll-like receptor (TLR) 4 agonist, was used as a control stimulus, as it is well known that it induces activation of APCs, including RAW 264.7 cells [43, 44]. The RAW 264.7 and JAWSII activation state was also determined by release of broad panel of cytokines and chemokines. An increase in the expression pattern of cell activation markers following VP6 exposure was accompanied by increase in proinflammatory cytokine secretion, primarily TNF-*α* by RAW cells and IL-6 by JAWSII cells. In RAW cells, 50 *μ*g/mL VP6 nanotubes induced TNF-*α* at a similar level to RV Wa and GII.4 VLPs stimulation. Interestingly, only VP6 nanotubes but not GII.4 VLPs or RV Wa stimulated remarkably high TNF-*α* and IL-6 response in JAWSII DCs. Consistent with this observation, Istrate et al. [45] reported that even if RV dlVLPs were internalized by DCs, they could not trigger their activation. A key proinflammatory cytokine, TNF-*α*, recruits and activates APCs at the site of inflammation (antigen delivery) and facilitates APC migration to the lymph nodes [46], thereby improving the antigen uptake and presentation to MHC I and MHC II restricted T cells. In addition, TNF-*α* and IL-6 have important roles in the generation of B-cell functions like proliferation and antibody secretion [47]. Overall, quantification of TNF-*α* and IL-6 showed that VP6 nanotubes are more potent in inducing these cytokines than NoV VLPs or RV. In addition, a nonpolymeric VP6 protein was not able to induce cytokine secretion near to the VP6 nanotubes. These results underline the importance of intact high order tubular structure conformation of VP6 in activation of APCs.

Furthermore, changes in the levels of different cytokines and chemokines were evaluated in untreated or VP6 stimulated RAW 264.7 cells by cytokine array. The results suggested that VP6 induces multiple cytokines (TNF-*α*, G-CSF, and IL-1ra) and chemokines (IP-10, MIP-1*β*, MIP-2, and RANTES) secretion into the supernatants of stimulated cells. G-CSF is a multifunctional cytokine [48] that is required for modulating macrophage and DC responses [38] and T cell responses following antigen stimulation [49]. RANTES, IP-10, IL-8, MIP-1*β*, and monocyte chemoattractant protein-1 (MCP-1) have been suggested to be the key host factors in gastrointestinal immunity during RV infection [50, 51].

Employing highly purified protein preparations is of high importance when investigating adjuvant properties of a protein, eliminating the immunostimulatory effect of impurities related to the protein expression system on APCs. The results with VLP preparations containing residual BV particles have strongly suggested that BV contaminants can trigger an innate immunity, inducing inflammatory cytokine production such as TNF-*α*, IL-6, and IFN-*α* [44, 52, 53]. Therefore, we tested RAW 264.7 and JAWSII cells for secretion of IFN-*α*, an antiviral cytokine induced upon live virus or virus-derived dsRNA stimulation of the cells through TLR3 [45, 53, 54]. Only RV Wa stimulation but not any other stimuli including highly purified VP6 nanotubes, GII.4 VLP, or LPS induced significant release of the cytokine by these cell lines showing that the recombinant proteins used to stimulate the APC in our assays were free of BV contamination.

The size from 0.2 *μ*m to 1.5 *μ*m and the morphology of VP6 nanotubes are favorable for macrophages and DC recognition and uptake resembling those of microorganisms [30, 31, 55]. Antigen acquisition by APCs is mediated by several uptake mechanisms such as receptor-mediated endocytosis, macropinocytosis, and phagocytosis, depending of the nature of the antigen [56–59]. Smaller particles (20–200 nm) are usually internalized via receptor-mediated endocytosis dependent on membrane clathrin or caveolin [60]. Larger particles are taken up by macrophages, B cells, Langerhans cells, and DC, through receptor-independent macropinocytosis (>0.5 *μ*m) or receptor-mediated phagocytosis (0.5–5 *μ*m) [61]. Bluetongue virus nonstructural NS1 protein tubular structures, which are similar in size (up to 1 *μ*m long and app. 50 nm in diameter) and morphology to VP6 nanotubes, were suggested to be internalized by macrophages and DC via endocytosis and macropinocytosis [62]. We used chemical inhibitors M*β*CD, chloroquine, and cytochalasin D to study the possible uptake and activation pathways of VP6 nanotubes [63]. TNF-*α* secretion was used for evaluating inflammatory APC activation and impure VP6 preparation containing production related impurities, including BV, was included as a control. Our results indicated a difference in the mechanism of RAW 264.7 cells uptake and activation by the two RV VP6 protein preparations. M*β*CD treatment depletes cholesterol which is a reversible mechanism that inhibits the formation of cholesterol-rich microdomains termed lipid rafts [64] important in the lipid raft-mediated endocytosis. Interestingly, when pure VP6 nanotubes were used, M*β*CD partially inhibited TNF-*α* secretion (22%), whereas impure VP6-induced TNF-*α* secretion was blocked almost completely by clathrin-dependent endocytosis inhibitor chloroquine. In support to our findings, Rodríguez et al. [31] showed by fluorescent microscopy the inhibition of VP6 cellular entry by M*β*CD. Furthermore, Abe et al. [53] observed that treatment with chloroquine inhibited BV-induced innate immune system activation and TNF-*α* secretion through BV gp64-mediated membrane fusion and TLR9/MyD88-dependent pathway. Cytochalasin D, which inhibits actin-dependent cellular processes such as phagocytosis [65], was not observed to have an effect on TNF-*α* secretion or VP6 protein uptake, unlike the published results where BV expression system-derived human immunodeficiency virus VLPs entry to APCs was blocked [66]. Altogether, the results with chemical inhibitors indicate that VP6 entry and activation of RAW cells require host cholesterol and may be partially mediated by lipid rafts. Due to the size range of VP6 nanotubes (~0.2–1.5 *μ*m, [30]), several entry pathways are likely to be involved and reaching total blocking is challenging.

Adjuvants generally work to spare the dose of particular vaccine antigen, to broaden the immune responses, and to prolong the duration of the response. The results of this study support our earlier findings of VP6 adjuvant effect on NoV VLPs immunogenicity* in vivo* [24] and show that VP6 nanotubes are efficiently internalized by APCs and induce activation and maturation of APCs. Induction of proinflammatory cytokines and chemokines by VP6 stimulated APCs, as shown in here, indicates efficient activation of the innate immune system, a major mechanism of adjuvant action. A depo effect is yet another important mechanism of adjuvant action that facilitates and improves the uptake of antigens by APCs. In here, a 30% increase in NoV GII.4 VLP uptake by APCs was observed when NoV VLPs were codelivered with RV VP6, compared to GII.4 VLP alone. In addition, VP6 coadministration showed increased activation and maturation of APCs measured by TNF-*α* production and upregulation of cell surface markers expression compared to NoV VLPs alone. Moreover, the exposure of RAW cells to GII.4 VLPs caused downregulation of MHC II expression, which was corrected by addition of VP6. Finally, our results identify the mechanisms of VP6 adjuvant action* in vitro*. VP6 nanotubes activate and mature APCs and increase the uptake of NoV VLPs by the APC.

## 5. Conclusions

In the present study, we show that recombinant RV inner core capsid VP6 nanotubes are efficiently internalized by professional APCs probably by a clathrin-independent endocytosis pathway involving cholesterol-rich lipid rafts, but likely other internalization routes are also involved. Moreover, VP6 nanotubes efficiently activate and mature macrophages and DCs as shown by upregulation of costimulatory molecules and MHC II expression and release of inflammatory mediators such as TNF-*α*, thus augmenting the action of cells of both innate and adaptive immune system. Furthermore, VP6 nanotubes facilitate the APC uptake of codelivered antigen, NoV GII.4 VLPs. These results provide insights into the mechanism of adjuvant action of VP6 nanotubes and confirm the immunostimulatory and immunomodulatory potential of VP6 observed* in vivo*.
